# Division of labor: multiple and specialized controls of vegetative growth and development in a poplar tree

**DOI:** 10.1186/1753-6561-5-S7-I10

**Published:** 2011-09-13

**Authors:** Victor Busov, Yiru Chen, Jiqin Gou, Steven Strauss, Yordan Yordanov, Christine Zawaski

**Affiliations:** 1 Michigan Technological University, USA; 2 Oregon State University, USA

## 

Plants size, shape and adaptability are determined in large by their capacity to sustain, spur, redirect or arrest localized and whole-plant growth. We show that GA signaling and metabolism determines the level and extent of shoot and root growth. Increase of GA concentrations or signaling leads to increased shoot growth but suppresses root development. These GA-related responses are underpinned by sets of highly specialized in their functions enzymes and signaling factors and cross-talk with other hormonal pathways. The differential effects of GA on root and shoot growth and development are likely associated with a regulatory mechanism responding to optimum and stress conditions. Ongoing work in the laboratory employs genomics and genetics approaches to more thoroughly understand poplar growth and development under stress conditions including nitrogen and water limitations. We use genetic networks analysis and forward genetics approaches to identify key regulators of poplar roots response to stress. Besides overall growth, trees show incredible repertoire of spatiotemporal regulation of growth in relation to control of organ size, growth periodicity and tropic responses. We have identified and characterized novel regulators of these processes in poplar.

